# Increase in direct costs for health systems due to lupus nephritis: the case of Colombia

**DOI:** 10.31744/einstein_journal/2022AO6553

**Published:** 2022-04-13

**Authors:** Sergio I Prada, Ana M Pérez, Ivana Nieto-Aristizábal, Gabriel J Tobón

**Affiliations:** 1 Fundación Valle del Lili Cali Colombia Fundación Valle del Lili, Cali, Colombia.; 2 University of Minnesota Minnesota United States University of Minnesota, Minnesota, United States.; 3 Universidad Icesi Cali Colombia Universidad Icesi, Cali, Colombia.

**Keywords:** Lupus nephritis, Direct service cost, Health systems, Lupus erythematosus, systemic, Therapeutics, Kidney failure, chronic, Health care costs, Colombia

## Abstract

**Objective:**

Lupus nephritis is one of the most severe complications of systemic lupus erythematosus and it has been estimated that can occur in up to 60% of patients. Direct costs of lupus nephritis have not been studied in developing countries. This study aimed to describe lupus nephritis direct costs in Colombia.

**Methods:**

Administrative data from two Colombian health maintenance organizations for 2014 and 2015 was obtained. An algorithm based on the International Statistical Classification of Diseases and Related Health Problems 10^th^ revision codes was developed to identify patients with lupus nephritis and lupus nephritis under study.

**Results:**

The average annual per-patient, all-claims, all-cause direct cost for lupus nephritis was US$ 12,624, 7.5 times higher than the average lupus patient without lupus nephritis. For lupus nephritis cases under study, estimated direct cost was US$ 3,664, 2 times higher than average lupus patient in Colombia. Difference in lupus nephritis patients is mainly accounted for the cost and frequency of procedures, exceeding by a factor of 5 the cost for durable medical equipment and the cost for drugs, respectively.

**Conclusion:**

Lupus patients who progress to lupus nephritis stage increased seven-fold the average annual per-patient, all-claims, and all-cause direct cost for the Colombian health system.

## INTRODUCTION

Systemic lupus erythematosus (SLE) is a chronic, autoimmune and systemic disease with the potential to affect any organ or system. It is well known that renal involvement, lupus nephritis, is the most severe complication due to its association with progression to end-stage renal disease (ESRD), and mortality.^(1)^ Lupus nephritis occurs in 38.5% of patients with recent diagnosis of SLE,^(2)^ in the first five years of the disease in the majority of cases^(3)^ and it has been described that 60% of SLE patients will develop the complication through the disease evolution.^(4,5)^ In a study conducted in SLE hospitalized patients, lupus nephritis was the most common organ involvement during 4 years.^(6)^

Regarding ethnicity it predominance has affected by 20% of black people and 52% of Asian Pacific Islanders.^(4)^ Specifically, in Colombia, the population is a mixture of black, white and indigenous peoples. This ethnicity confers higher risk of developing lupus nephritis as it has been demonstrated before.^(7,8)^ Also, lupus nephritis affects more men than women, and contrary to what has been seen in SLE without the complication,^(5,9,10)^ it is more often seen among younger patients.^(1,11,12)^ In pediatric patients, it occurs in 50-75% of them and the vast majority of them have developed the complication in the first two years of SLE diagnosis, and Hispanic population is one of the ethnicities at higher risk.^(13)^ However, the histological form of presentation of lupus nephritis has no significant differences compared with Caucasians.^(14)^

The confirmed diagnosis remains dependent on a renal biopsy, a procedure that is not only invasive but costly as well. It may also be needed during the course of the disease when severe flares occur or discard histological transformation.^(15)^ However, some approaches based on urinary biomarkers are being proposed to ameliorate these issues in the future.^(16)^

The main treatment strategies consist of an induction scheme of cyclophosphamide (or mofetil mycophenolate) followed by mofetil mycophenolate or other immunosuppressants for maintenance. In spite of adequate treatment, renal flares have an incidence of 27%- 66%.^(15)^

Due to its less favorable prognosis, lupus nephritis is associated with higher rates of hospitalization, procedures such as dialysis, and surgery in cases of renal transplantation.^(17,18)^

All the above mentioned healthcare resources, act as determinants of an increase in the direct costs of lupus nephritis.^(19)^ The literature on direct cost of these patients is concentrated in the developed world. A study in Canada with a cohort of 141 patients conducted between 2004 and 2009 found direct cost of outpatient services associated with SLE with lupus nephritis totalizing Canadian US$ 12,597 Canadian dollar compared with Canadian US$ 10,585 in those without lupus nephritis.^(20)^ A study in the United States (US) with 2,298 patients from 1999 to 2005, estimated that lupus nephritis had a cost of US$ 27,463 in the first year including all outpatient and inpatient healthcare services, after this period cost has increased.^(21)^ This study also showed that patients with lupus nephritis were more than twice as costly as patients without the disease. Another paper from the US with 15,590 patients showed that between 2006 and 2008, patients with lupus nephritis spent US$ 6,029 in pharmacy, US$ 15,267 in outpatient services, and US$ 9,292 in hospitalization. In contrast, patients without lupus nephritis had a cost of US$ 3,190; US$ 6,202 and US$ 2,636, respectively.^(22)^

The Colombian health system design follows the managed competition principles. The Ministry of Health defines the national benefits package and pays a per capita premium to health plans. Enrollment in health plans is mandatory, so health plans compete to attract members. Providers of healthcare are either private or public and also compete to be part of health plan’s network. The system is divided in two subsystems. One called “contributive” because it is financed by employer-employee contributions and covers the working population and their families. The other one is called “subsidized” because it is financed by the national government and cover the poor. There is a private insurance market in which people may buy additional coverage. Lupus treatment is partially covered by the national government-defined benefits package. Treatment beyond such coverage is obtained either by judicialization or by private insurance.

In a previous paper published by our group, annual direct costs of lupus care resulted in US$ 2,355, in average, per-patient, all-claims, and all-cause.^(23)^

## OBJECTIVE

We aimed to show the all-claims, all-cause cost of lupus nephritis patients for a health system for the first time in a developing country using administrative claims data.

## METHODS

### Subjects

This is a descriptive data study. We used administrative claims from two private health maintenance organizations (HMOs) for years 2014 and 2015. Data was obtained under an academic agreement between Fundación Valle del Lili and Universidad Icesi, Cali, Colombia and both organizations. As this is a study based on claims data, an Ethics Committee approval was not required. In 2015, together both HMOs insured around 4 million lives accounting for 17% of the national health insurance plan for the working population, and almost half of the enrollees in Colombia´s southwest region.^(23)^

Two datasets were linked by a unique identifier. The first database corresponds to claims paid for by these HMOs using funds received from the national government to cover the government-defined public benefits package. The second, claims of services did not include in the national public benefits package, but also paid for by the government using a reimbursement mechanism. This is a characteristic of the Colombian system by which high-cost drugs or procedures are allowed in case by case basis following judicial rulings.^(24,25)^

The public benefits package data includes demographics as well as services rendered. Demographics include: gender, age, city of residence, educational level, and enrolment types (primarily-insured or beneficiaries). Services rendered include: a unique patient identification, date when the service was provided, type of service (inpatient, outpatient, in-home, and emergency care), the mechanism of reimbursement (capitation or fee-for-service), municipality, primary diagnosis, service type (drug, procedures, durable medical equipment - DME), and the amount paid for by the insurer to the provider. The non- public benefits package data include: patient identification, service date, amount reimbursed, and service type (drugs, procedures, and DME).

### Inclusion criteria

Lupus patients were selected using the tenth revision of the International Statistical Classification of Diseases and Related Health Problems 10^th^ (ICD-10). Diagnosis included were: discoid lupus erythematosus (DLE), L930; subacute cutaneous lupus erythematosus, L931; drug-induced SLE, M320; SLE with organ or system involvement, M321; other forms of SLE, M328; and unspecified SLE, M329. After excluding the patients that did not have the diagnosis in both years (2014 and 2015), the final sample was 3,592 patients.^(23)^

**Figure 1:**
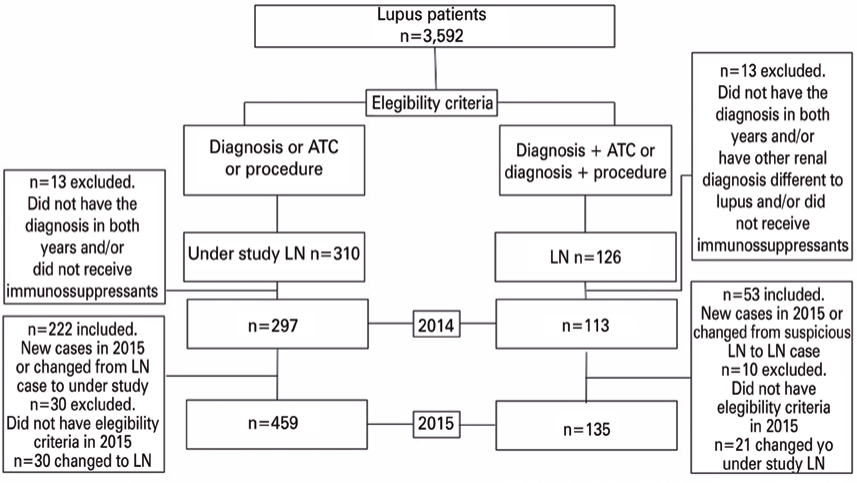
Algorithm for ion of cases

LN: lupus nephritis; ATC: anatomical therapeutic chemical.

To identify lupus nephritis patients, we developed an algorithm using specific rules for diagnoses, drugs, and procedures information as present in the datasets. First, the ICD-10 was used for this purpose. The following diagnoses were included as indicators of the disease (Figure 1):

- Nephrotic syndrome with other morphologic changes, N048;
- Acute kidney failure with tubular necrosis, N170;
- Acute kidney failure with acute cortical necrosis, N171;
- Other acute kidney failure, N178;
- Acute kidney failure, unspecified, N179;
- Unspecified kidney failure, N19;
- End-stage renal disease, N180;
- Other chronic kidney diseases, N188;
- Chronic kidney disease, unspecified, N189;
- Kidney transplant status, Z940.

International Statistical Classification of Diseases and Related Health Problems 10^th^ (ICD-10).

Second, we used the record of outpatient drugs paid for by the insurer associated with the unique ID of the patient during the period of the study. The Anatomical Therapeutic Chemical (ATC) classification system was used. Codes that were considered highly likely associated with lupus nephritis are the following:

- Cyclophosphamide, L01AA01;
- Mycophenolic acid, L04AA06.

Third, the following CD-10 revision procedure classification system were included:

- Kidney biopsy;
- Kidney transplant;
- Hemodialysis;
- Peritoneal dialysis.

Patients were classified as lupus nephritis if they had either a diagnosis and underwent a related procedure, or had a diagnosis and were treated with one of the listed drugs. However, not all patients coincided perfectly, filling only one criterion or the other. To avoid discarding likely cases of lupus nephritis, a category called “suspected lupus nephritis” was included in the study, composed of patients identified by one method. The classification algorithm was applied to years 2014 and 2015. Patients that were identified in 2014, but not in 2015, were revised in detail by one of the authors, a practitioner rheumatologist who studied every claim of those patients. After the rheumatologist analysis, 23 false positive cases of lupus nephritis were excluded, because they presented additional diagnoses to which renal involvement was attributable, such as toxic nephropathy, or were not taking any immunosuppressant drugs. The number of patients with lupus nephritis in 2014 and 2015 were 113 and 135, respectively. The cases of suspected lupus nephritis were 297 and 459, respectively (Figure 1).

## COST

Direct cost for the government funded Colombian health system are defined as payments made by insurers to providers. Cost was estimated by summing all-cause public benefits package and non-public benefits package services rendered and paid for lupus nephritis patients. The official currency of Colombian is the peso (COP), however, in the results section figures are in dollars for comparability with the international literature. We used the average exchange rate from both years (2,372 COP per 1 US$) to convert from COP to US$. Annual all-cause all-claims direct cost from public benefits package was calculated by type of service (drugs, procedures, and DME), point of service (urgent care, inpatient, outpatient, and in-home). No statistical inference tests were performed between years because the data is not a sample but a population of patients for both insurers.

## RESULTS

In table 1, direct average all-cause and all-claims cost per patient with lupus nephritis is shown by gender and patient classification. Average annual per-patient, all claims, all-cause direct cost for lupus in general was reported elsewhere.^(23)^ Patients with lupus nephritis were 7,46 times as costly as patients without lupus nephritis in 2015. Suspected-lupus nephritis patients were 2,16 times as costly as patients without lupus nephritis in 2015.

**Table 1:** Average direct cost for lupus and lupus nephritis patients by gender and type

Costs by gender and type	n		Mean ($)		SD		
		
	2014	2015	2014	2015	Change (%)	2014	2015
All	3,592	3,592	2,163	2,355	8.9	5,496	5,674
By gender							
Men	356	356	2,362	2,253	- 4.6	4,760	4,363
Women	3,236	3,236	2,141	2,366	10.5	5,572	5,800
By type							
No LN	3,182	2,998	1,602	1,692	5.6	3,673	3,868
LN	113	135	11,405	12,624	10.7	15,220	17,244
LN under study	297	459	4,656	3,664	- 21.3	9,574	5,642

LN: lupus nephritis; SD: standard deviation.

Table 2 gives direct cost paid for by insurers per patient only for lupus nephritis patients by type of service, point of service and by type of expenditure. By type of service procedures are the most frequent and the more expensive claims per patient. Procedures cost 5.7 times as drugs and 4.9 times as DME. By point of service outpatient and inpatient visits are the most frequent claims by patient. Non-PBP services comprise 31% and 25% of total direct cost for 2014 and 2015, respectively.

**Table 2:** Average direct cost per patient with lupus nephritis by type and point of service

Costs by type and point of service	n		Mean ($)			SD		
		
	2014	2015	2014	2015	Change (%)	2014		2015
PBP by type of service								
Drugs	110	133	1,312	1,411	7.6	2,091		2,801
DME	84	81	1,211	1,531	26.4	5,502		5,306
Procedures	113	135	6,433	7,542	17.2	8,219		10,935
PBP by point of service								
Urgent care	89	90	221	323	46.2	275		517
Outpatient	113	135	2,886	4,651	61.2	3,997		4,264
Inpatient	103	100	6,077	6,704	10.3	10,883		14,358
In-home	10	8	120	303	152.8	300		461
Average direct cost per patient user	113	135	8,610	9,851	14.4	11,388		15,154
Non-PBP	89	116	3,548	3,228	-9.0	9,534		8,369
PBP and Non PBP	113	135	11,405	12,624	10.7	15,220		17,244

SD: standard deviation; PBP: public benefit package; DME: durable medical equipment.

Table 3 direct cost paid for by insurers per patient only for suspected-lupus nephritis patients by type of service, point of service and by type of expenditure. Results are similar to lupus nephritis patients, but monetary outlays are between 30% and 40% of confirmed cases.

**Table 3:** Average direct cost per patient with lupus nephritis under study by type and point of service

	n		Mean ($)			SD	
	2014	2015	2014	2015	Change (%)	2014	2015
PBP by type of service							
Drugs	286	456	596	587	-1.6	1,915	2,224
DME	134	203	402	328	-18.4	1,123	1,314
Procedures	294	455	2,328	1,592	-31.6	4,231	2,507
PBP by point of service							
Urgent care	150	246	147	124	-15.8	200	197
Outpatient	297	459	1,055	1,459	38.3	1,766	2,439
Inpatient	195	255	2,921	1,394	-52.3	5,769	3,343
In-home	23	21	162	130	-19.8	287	157
Average direct cost per patient user	297	459	3,059	2,306	-24.6	5,334	4,011
Non-PBP	220	343	2,155	1,818	-15.6	7,265	3,968
PBP and non PBP	297	459	4,656	3,664	-21.3	9,574	5,642

SD: standard deviation; PBP: public benefit package; DME: durable medical equipment.

Table 4 and 5 show the top 10 procedures sorted by cost per procedure in both lupus nephritis and suspected-lupus nephritis in 2014 and 2015. For lupus nephritis, the top 3 procedures were nephrectomy, renal transplantation and intensive care unit (ICU) hospitalization; while for suspected-lupus nephritis the top 4 procedures were ICU hospitalization, intermediate unit care hospitalization, renal biopsy and hospitalization in moderate/high complexity services.

**Table 4:** Cost per procedure for Lupus nephritis patients (more costly procedures)

Procedure	n (Procedure)	Mean	SD	Procedure	n (Procedure)	Mean	SD
	
	2014				2015		
Nephrectomy	3	4,201	5,014	Renal transplantation	8	6,085	4,710
ICU hospitalization	29	2,897	1,677	ICU hospitalization	16	5,259	4,093
Renal transplantation	13	2,716	2,658	Nephrectomy of transplanted or rejected kidney	1	1,227	
Intermediate care unit hospitalization	25	1,027	594	Hospitalization in moderate-complexity service	54	992	856
Peritoneal dialysis	38	977	108	Hospitalization in high-complexity service	49	885	1,230
Partial hospitalization/Day hospital	2	762	180	Hospitalization in low-complexity service	67	745	771
Hospitalization in moderate-complexity service	111	680	591	Hemodialysis	414	713	371
Hospitalization in low-complexity service	115	614	544	Class I and II HLA test	10	655	153
Class I and II HLA test	12	451	228	Intermediate care unit hospitalization	5	537	374
Renal biopsy	30	439	398	Class I HLA test	1	519	

SD: standard deviation; ICU: intensive care unit; HLA: histocompatibility leucocyte antigen.

**Table 5:** Cost per procedure for under study- lupus nephritis patients (more costly procedures)

Procedure	n (Procedure)	Mean	SD	Procedure	n (Procedure)	Mean	SD
	
	2014				2015		
ICU hospitalization	25	2,496	1,645	ICU hospitalization	10	4,716	3,492
Intermediate care unit hospitalization	16	1,893	1,578	Renal biopsy	27	660	525
Hospitalization in high-complexity service	44	592	498	Hospitalization in moderate-complexity service	84	595	490
Hospitalization in low-complexity service	146	559	534	Therapeutic plasma exchange	1	584	
Renal biopsy	32	510	380	Hospitalization in high-complexity service	87	553	531

SD: standard deviation; ICU: intensive care unit; HLA: histocompatibility leucocyte antigen.

## DISCUSSION

This paper described all-cause all-claims direct costs paid for by insurers associated with lupus nephritis patients, based on administrative data of patients enrolled for two consecutive years, 2014 and 2015, in two HMOs in Colombia. Of 3,592 patients with lupus diagnosis, we developed an algorithm to identify patients with lupus nephritis under study and lupus nephritis, that accounted for 523 and 594 patients in 2014 and 2015, respectively.

We found that all-cause average annual direct costs for these patients to be US$ 11,405 and US$ 12,624 in 2014 and 2015,respectively. Patients with lupus nephritis were 7.46 times as costly as patients without lupus nephritis in 2015. The Lupus erythematosus Cost of Illness in Europe (LUCIE) study evaluated healthcare costs of lupus in 427 patients of five European countries, and reported that one of the predictors of higher costs was renal involvement, adding US$ 711 to the costs.^(26)^ In Australia, Yeo et al. evaluated 200 patients with lupus from 2013 until 2016, and found that the mean annual direct costs was of US$ 7,413, and again, renal involvement was the organ manifestation that annually incremented costs the majority in 62.5%.^(27)^ Tanaka et al. studied 295 Japanese patients with lupus from 2010 to 2012, with a mean cost during that period of time of US$ 27,004. They also they divided the disease severity in mild, moderate and severe, for which the costs were US$ 5,549, US$ 15,290, and US$ 43,322, respectively. In these groups, patients with renal manifestations had 78.3% greater costs than those without this disease.^(28)^

Regarding type of services, we found that for both lupus nephritis and lupus nephritis under study, procedures were the most frequent and costly expense for insurers. By the point of service, inpatient services for lupus nephritis and lupus nephritis under study were the most important in both years, except for lupus nephritis under study in 2015 that was outpatient services. More specifically for lupus nephritis, the most expensive healthcare services per procedure rendered were surgical procedures such as nephrectomy and renal transplantation, while for lupus nephritis under study, it was renal biopsy; for both high complexity hospitalization services including ICU stays. The lack of clinical data prevents us to confirm severity, but costs by type/point of services, are likely associated with patients in more advanced stages or with higher number of comorbidities, which would be proportionally associated with higher all-cause and all-claims costs per-patient.

In a similar way, a multicentric study that included patients from the US, Mexico, Korea, Canada and Europe found that in patients with lupus nephritis and with GFR <30mL/minute or ESRD, the dialysis was the most expensive type of service whereas in patients without lupus nephritis, the most expensive issue was the drugs. Also, according to this study, the worse the renal function the higher the five and ten-year cumulative costs as in patients with ESRD that were expected to cost 23-fold that of non-lupus nephritis patients.^(29)^ Furst et al. compared 907 lupus nephritis patients with controls in the USA, and reported that 98.90% of patients used diagnostic services annually.^(30)^ Renal biopsy as a mandatory procedure to confirm prognosis and sometimes to follow-up,^(15)^ surely contributes to the finding of this study.

A recent study showed that mortality in Latin population was four times higher than expected.^(31)^ Lupus nephritis is the main SLE complication associated with death, as well as lupus nephritis patients are at high risk of developing ESRD.^(32)^ In this way, management guidelines emphasize that the aims of treating lupus nephritis are to favor patients survival, to avoid flares and to preserve kidney function, among others. All of which may be accomplished when accessing multidisciplinary healthcare attention whenever possible.^(33)^

### Study limitations

Our study is based on administrative data, thus accuracy may be affected by the lack of clinical data.

## CONCLUSION

Lupus patients that progressed to lupus nephritis stage showed seven-fold increase of the average annual per-patient, all-claims, all-cause direct cost for the Colombian health system. Lupus nephritis is associated with high morbidity and mortality, adequate access to early and periodical multidisciplinary management that could improve outcomes, and therefore, costs related with complications such as renal replacement therapy and transplant.

## References

[1] Hanly JG, O’Keeffe AG, Su L, Urowitz MB, Romero-Diaz J, Gordon C, et al. The frequency and outcome of lupus nephritis: Results from an international inception cohort study. Rheumatology (Oxford). 2016;55(2):252-62. 10.1093/rheumatology/kev311 PMC4939728 26342222

[2] La Paglia GM, Leone MC, Lepri G, Vagelli R, Valentini E, Alunno A, et al. One year in review 2017: systemic lupus erythematosus. Clin Exp Rheumatol. 2017;35(4):551-61. Review. 28721860

[3] Borchers AT, Leibushor N, Naguwa SM, Cheema GS, Shoenfeld Y, Gershwin ME. Lupus nephritis: a critical review. Autoimmun Rev. 2012;12(2):174-94. Review. 10.1016/j.autrev.2012.08.018 22982174

[4] Drenkard C, Lim SS. Update on lupus epidemiology: advancing health disparities research through the study of minority populations. Curr Opin Rheumatol. 2019;31(6):689-96. Review 10.1097/BOR.0000000000000646 PMC6791519 31436582

[5] Anders HJ, Saxena R, Zhao M, Parodis I, Salmon JE, Mohan C. Lupus nephritis. Nat Rev Dis Primers. 2020;6(1):7. Review. 10.1038/s41572-019-0141-9 31974366

[6] Tan Y, Yu F, Long J, Gan L, Wang H, Zhang L, et al. Frequency of systemic lupus erythematosus was decreasing among hospitalized patients from 2013 to 2017 in a National Database in China. Front Med (Lausanne). 2021;8:648727. 10.3389/fmed.2021.648727 PMC8056078 33889586

[7] Pons-Estel GJ, Alarcón GS, Hachuel L, Boggio G, Wojdyla D, Pascual-Ramos V, Soriano ER, Saurit V, Cavalcanti FS, Guzman RA, Guibert-Toledano M, Sauza Del Pozo MJ, Amigo MC, Alva M, Esteva-Spinetti MH, Pons-Estel BA; GLADEL. Anti-malarials exert a protective effect while Mestizo patients are at increased risk of developing SLE renal disease: data from a Latin-American cohort. Rheumatology (Oxford). 2012;51(7):1293-8. 10.1093/rheumatology/ker514 PMC3380245 22389125

[8] Pons-Estel GJ, Alarcón GS, Burgos PI, Hachuel L, Boggio G, Wojdyla D, Nieto R, Alvarellos A, Catoggio LJ, Guibert-Toledano M, Sarano J, Massardo L, Vásquez GM, Iglesias-Gamarra A, Lavras Costallat LT, Da Silva NA, Alfaro JL, Abadi I, Segami MI, Huerta G, Cardiel MH, Pons-Estel BA; Grupo Latino Americano de Estudio de Lupus (GLADEL). Mestizos with systemic lupus erythematosus develop renal disease early while antimalarials retard its appearance: data from a Latin American cohort. Lupus. 2013;22(9):899-907. 10.1177/0961203313496339 PMC3943422 23857989

[9] Schwartzman-Morris J, Putterman C. Gender differences in the pathogenesis and outcome of lupus and of lupus nephritis. Clin Dev Immunol. 2012;2012:604892. Review. 10.1155/2012/604892 PMC3368358 22690240

[10] Somai M, Daoud F, Rachdi I, Zoubeidi H, Raies L, Aydi Z, et al. Predictive factors of the lupus nephritis in a Tunisian cohort. Tunis Med. 2019;97(12):1399-406. 32173811

[11] Mahmoud SS, Bazaraa HM, Lotfy HM, Abd-El-Aziz DM. Renal involvement in childhood-onset systemic lupus erythematosus in Egypt. Rheumatol Int. 2012;32(1):47-51. 10.1007/s00296-010-1554-7 20658237

[12] Seligman VA, Lum RF, Olson JL, Li H, Criswell LA. Demographic differences in the development of lupus nephritis : a retrospective analysis. Am J Med. 2002;112(9):726-9. 10.1016/s0002-9343(02)01118-x 12079714

[13] Pinheiro SV, Dias RF, Fabiano RC, Araujo SA, Silva AC. Pediatric lupus nephritis. J Bras Nefrol. 2019;41(2):252-65. Review. 10.1590/2175-8239-JBN-2018-0097 PMC6699445 30465590

[14] Fiorot FJ, Islabão AG, Pereira RM, Terreri MT, Saad-Magalhães C, Novak GV, Molinari BC, Sakamoto AP, Aikawa NE, Campos LM, Peracchi OA, Appenzeller S, Ferriani VP, Silva MF, Fonseca AR, Sztajnbok FR, Paim LB, Fraga MM, Okuda EM, Bica BE, Sena EG, Moraes AJ, Rolim AM, Spelling PF, Scheibel IM, Cavalcanti AS, Matos EN, Robazzi TC, Guimarães LJ, Santos FP, Ramos VC, Carneiro-Sampaio M, Bonfá E, Silva CA; Brazilian Childhood-onset Systemic Lupus Erythematosus Group. Disease presentation of 1312 childhood-onset systemic lupus erythematosus: influence of ethnicity. Clin Rheumatol. 2019;38(10):2857-63. 10.1007/s10067-019-04631-0 31209708

[15] Sidiropoulos PI, Kritikos HD, Boumpas DT. Lupus nephritis flares. Lupus. 2005;14(1):49-52. Review. 10.1191/0961203305lu2059oa 15732288

[16] Aguirre-Valencia D, Ríos-Serna LJ, Posso-Osorio I, Naranjo-Escobar J, López D, Bedoya-Joaqui V, et al. Expression of BAFF, APRIL, and cognate receptor genes in lupus nephritis and potential use as urinary biomarkers. J Transl Autoimmun. 2019;3:100027. 10.1016/j.jtauto.2019.100027 PMC7388398 32743512

[17] Jorge A, Wallace ZS, Lu N, Zhang Y, Choi HK. Renal transplantation and survival among patients with lupus nephritis: a cohort study. Ann Intern Med. 2019;170(4):240-7. 10.7326/M18-1570 PMC6739121 30665236

[18] Katalinić L, Eliasson E, Bubić-Filipi L, Kes P, Anić B, Basić-Jukić N. Renal transplantation in patients with lupus nephritis. Lijec Vjesn. 2014;136(7-8): 219-23. Review. 25327010

[19] Turchetti G, Yazdany J, Palla I, Yelin E, Mosca M. Systemic lupus erythematosus and the economic perspective: a systematic literature review and points to consider. Clin Exp Rheumatol. 2012;30(4 Suppl 73):S116-22. Review. PMC3714226 23072767

[20] Aghdassi E, Zhang W, St-Pierre Y, Clarke AE, Morrison S, Peeva V, Landolt-Marticorena C, Su J, Reich H, Scholey J, Herzenberg A, Pope JE, Peschken C; LuNNET CaNIOS Investigators, Wither JE, Fortin PR. Healthcare cost and loss of productivity in a Canadian population of patients with and without lupus nephritis. J Rheumatol. 2011;38(4):658-66. 10.3899/jrheum.100482 21159829

[21] Li T, Carls GS, Panopalis P, Wang S, Gibson TB, Goetzel RZ. Long-term medical costs and resource utilization in systemic lupus erythematosus and lupus nephritis: a five-year analysis of a large medicaid population. Arthritis Rheum. 2009;61(6):755-63. 10.1002/art.24545 19479688

[22] Pelletier EM, Ogale S, Yu E, Brunetta P, Garg J. Economic outcomes in patients diagnosed with systemic lupus erythematosus with versus without nephritis: results from an analysis of data from a US claims database. Clin Ther. 2009;31(11):2653-64. 10.1016/j.clinthera.2009.11.032 20110008

[23] Prada SI, Perez AM, Nieto-Aristizábal I, Tobón GJ. Direct cost of lupus care in the developing world: the case of Colombia. Lupus. 2019;28(8):970-6. 10.1177/0961203319856093 31204587

[24] Andia T, Lamprea E. Is the judicialization of health care bad for equity? A scoping review. Int J Equity Healt. 2019;18(1):61. Review. 10.1186/s12939-019-0961-y PMC6545687 31155005

[25] Lamprea E. The judicialization of health care: a global south perspective. Annu Rev Law Soc Sci. 2017;13:431-49.

[26] Doria A, Amoura Z, Cervera R, Khamastha MA, Schneider M, Richter J, et al. Annual direct medical cost of active systemic lupus erythematosus in five European countries. Ann Rheum Dis. 2014;73(1):154-60. 10.1136/annrheumdis-2012-202443 23264339

[27] Yeo AL, Koelmeyer R, Kandane-Rathnayake R, Golder V, Hoi A, Huq M, et al. Lupus Low disease activity state and reduced direct health care costs in patients with systemic lupus erythematosus. Arthritis Care Res (Hoboken). 2020;72(9):1289-95. 10.1002/acr.24023 31282076

[28] Tanaka Y, Mizukami A, Kobayashi A, Ito C, Matsuki T. Disease severity and economic burden in Japanese patients with systemic lupus erythematosus: a retrospective, observational study. Int J Rheum Dis. 2018;21(8):1609-18. 10.1111/1756-185X.13363 PMC6585770 30146745

[29] Barber MR, Hanly JG, Su L, Urowitz MB, St Pierre Y, Romero-Diaz J, et al. Economic evaluation of lupus nephritis in the systemic lupus international collaborating clinics inception cohort using a multistate model approach. Arthritis Care Res (Hoboken). 2018;70(9):1294-302. 10.1002/acr.23480 PMC6155450 29193883

[30] Furst DE, Clarke A, Fernandes AW, Bancroft T, Gajria K, Greth W, et al. Medical costs and healthcare resource use in patients with lupus nephritis and neuropsychiatric lupus in an insured population. J Med Econ. 2013;16(4):500-9. 10.3111/13696998.2013.772058 23363329

[31] Gianfrancesco MA, Dall’Era M, Murphy LB, Helmick CG, Li J, Rush S, et al. Mortality among minority populations with systemic lupus erythematosus, including Asian and Hispanic/Latino Persons -California, 2007-2017. MMWR Mor Mortal Wkly Rep. 2021;70(7):236-9. 10.15585/mmwr.mm7007a2 PMC7891689 33600382

[32] Ocampo-Piraquive V, Nieto-Aristizábal I, Cañas CA, Tobón GJ. Mortality in systemic lupus erythematosus: causes, predictors and interventions. Expert Rev Clin Immunol. 2018;14(12):1043-53. Review. 10.1080/1744666X.2018.1538789 30338717

[33] Fanouriakis A, Kostopoulou M, Cheema K, Anders HJ, Aringer M, Bajema Iet al. , 2019 Update of the Joint European League Against Rheumatism and European Renal Association-European Dialysis and Transplant Association (EULAR/ERA-EDTA) recommendations for the management of lupus nephritis. Ann Rheum Dis. 2020;79(6):713-23. 10.1136/annrheumdis-2020-216924 32220834

